# Family medicine ethical issues regarding physician-patient interactions from patients' perspectives: A qualitative study

**DOI:** 10.22088/cjim.12.2.184

**Published:** 2021-03

**Authors:** Seyyed Javad Madani, Bagher Larijani, Saharnaz Nedjat, Alireza Bagheri

**Affiliations:** 1 Medical Ethics and History of Medicine Research Center, Tehran University of Medical Sciences, Tehran, Iran; 2 Endocrinology and Metabolism Research Center, Endocrinology and Metabolism Clinical Sciences Institute, Tehran University of Medical Sciences, Tehran, Iran; 3 Department of Medical Ethics, School of Medicine, Tehran, University of Medical Sciences, Tehran, Iran; 4 Department of Epidemiology and Biostatistics, School of Public Health, Tehran University of Medical Sciences, Tehran, Iran; 5 Faculty of Medicine, Department of Medical Ethics, Mazandaran University of Medical Sciences, Sari, Iran; 6 Iran Knowledge Utilization Research Center, Tehran University of Medical Sciences, Tehran, Iran

**Keywords:** Ethical analysis, Family practice, Iran, Qualitative research, Ethics

## Abstract

**Background:**

The physician-patient relationship is important because the patient's satisfaction affects trust in physician and accepting physician's recommendations in medical treatment decisions. Understanding a patient’s opinion about a trustworthy and friendly physician as well as ethical issues regarding family medicine, therefore, gains double importance. This paper attempts to provide a comprehensive evaluation of the subject.

**Methods:**

In summer 2018, a conventional qualitative content analysis was done on 21 participants who were referred to family physicians in the North of Iran. Data were collected by means of purposive sampling and semi-structured face to face individual interviews. Participants shared their experiences about ethical considerations in family medicine. All interviews were recorded and transcribed word for word, data were analyzed using qualitative content analysis.

**Results:**

Data analysis resulted in the extraction of 7 categories and 21 sub-categories from the 71 initial codes. The categories include "responsibility", "patient's privacy", "informed consent", "respect and dignity of patient", "effective physician-patient communication", "trust in physician" and "conflict of interests".

**Conclusion:**

There are some differences between the participants' perceptions of ethical considerations in family medicine and opinions of medical ethics curriculum designers in Iran and particularly in the world. Some shared elements including "resource allocation", "the beginning and end of life", "research ethics", "substitute decision-making", etc. – all of them are main titles of ethics curriculum – could not be weighed as major ethical issues from the patients' perspectives. The patient's satisfaction and, therefore, the enhancement of mutual trust is essential. Patients’ comments should be considered when providing ethical guidelines.

Modernism and technology both have led to enormous changes in medicine, particularly after the specialization of medical fields. The number of specialists grew gradually and tendency towards public medicine decreased, and simultaneously, the need for generalists capable of providing comprehensive and continuous care increased (1-4). At this time, the need for a doctor with a different and somewhat traditional, but in fact, postmodern approach to medicine was felt which was called the family physician (5). Family medicine care is an accessible, comprehensive, contextual, community-based, and patient-centered communication-based model (6). 

Medicine, in general, is an attempt to preserve, promote and restore human health. But there are fundamental differences between modern medicine and family medicine. Family physicians are comprehensive doctors and focus on all aspects of human health, with special interest and anxiety. They consider physiological, psychological, social, environmental and existential aspects, not only biomedical issues (7-10). Therefore, ethics in family medicine vary significantly with ethics in other fields of medicine (7). 

This increases the importance of recognizing the issues of family medicine ethics, in order to design a specific ethical guideline, the Worldwide Organization of Family Doctors –WONCA – has emphasized implementing the ethical educational curriculum for professors and students of the family medicine as a primary goal (11). There are few family medicine ethics curriculum around the world, designed for other countries with different cultures, where the views of patients are largely neglected (12-15). In the review of literature, there was no study on the views of patients about the ethical considerations of this field (16).

This study was designed to examine the views of the recipients of the services on the ethical considerations of family medicine. Because, on the one hand, the implementation of the family medicine plan in Iran does not have a long history and the environmental, cultural, social and religious differences with countries such as the UK, Canada, United States, and others which are about 70 years of family practice. It does not seem that the ethical issues of this field in those countries are exactly our problems. On the other hand, as mentioned earlier, family physician-patient communication is of great importance. Because patient satisfaction plays an important role in trusting the doctor and accepting his guidance, the need to understand the patient's view of a good, ethical and reliable family physician and the ethical considerations involved in this relationship is felt more than ever. Prior to this article, there has been no study of patients' views on family medicine ethics in Iran. And no initial information is available. For this reason, qualitative content analysis was used to discover the ethical issues of family medicine from the patients’ perspective inductively.

**Methodology: **In summer 2018, a qualitative study conducted on the basis of in-depth semi-structured face to face individual interviews with participants attending family physicians in urban and rural environments in the North of Iran.

This article is the result of a part of a PhD thesis entitled "Ethical issues of family medicine program and preparing a preliminary version of ethical guideline for family medicine program in Iran".

**Sampling and collecting data:** Various urban and rural family health centers were searched for interviewees, and after explaining the study summary to the public, the enthusiastic participant's call number was received, after contacting them and receiving basic demographic information, purposive sampling was used to cover a wide range of patients with different ages, genders, educational levels, occupations, and social classes. The appropriate people were contacted for interviews, and face to face interviews were arranged at the time and place of their interest. The interviews began with the help of a questionnaire guide and ended after reaching data saturation. At the interview, the first author and another person were present as assistants. Interviews were recorded using a tape recorder. During the interviews, the first author wrote down what he thought on paper to serve as a memo to the analysis. Each interview was fully transcribed and primary coded based on content. The text of the interviews was submitted to the interviewees for validation, of which 70% were approved.

Interview questions included: 1) physician behavior when visiting a patient, 2) how to communicate with the doctor and level of trust in him/her, and 3) the profile of a trustworthy and friendly family doctor. For more information and more comprehensive answers, additional explanations are provided, along with some more in-depth questions to better understand the subject. Then, by asking clear and definitive questions, the interview process was guided to cover the purpose of the research, the continuation of the interview largely relied on questions that spontaneously arose in the interviewer and interviewee interaction. The interviewees were asked to share their experiences on the various issues including the behavior of the physician toward their family, the environment, the patient's visiting process, as well as the level of interaction and mutual trust. They were then asked to compare their experiences with the time before the program and highlight the ethical and/or non-ethical aspects of the subject. For these explanations and questions, we used family medicine's philosophy and principles and family medicine ethics curriculum in different countries (4, 6, 12-15).A total of 21 interviews were conducted, each lasted for 20 to 55 minutes (35 minutes on average). 

**Content analysis:** After the interview, their content analysis was carried out using a conventional content analysis approach (17). The interviews were written word-by-word to extract the baseline codes, the interviews were read carefully and repeatedly to obtain a general understanding of the data and the words or phrases that indicated the key thoughts or concepts of the data were identified. Then the codes were transformed into subcategories that were more abstract than the initial codes based on their similarities and differences. Based on the existing relationships and differences and similarities, the extracted sub-categories were combined and categorized and all categories were extracted which is evident in table 2.

**Trustworthiness:** To ensure the scientific validity of the qualitative study, Lincoln and Guba's four criteria, which include acceptance, reliability, portability, and acceptability, were used (18). Also, the transcripts of the interviews and extracted codes were provided to the professors of the research team and to two individuals who were dominant in the qualitative research and their suggestions were implemented in the coding process. The first researcher tried to increase the validity of the data by prolonged involvement with the phenomenon and complete immersion in the data. Data analysis was also provided to a number of qualitative researchers. Comparisons were made between the results of the study and those that needed correction. To confirm the study's acceptability, the researchers attempted to accurately record and report the research stages during the study to enable others to carry out the study if needed. To provide portability, the researchers sought to provide the necessary context for judging and evaluating others by accurately describing the research.

**Ethical considerations:** All participants received full explanations about the study objectives and the terms of confidentiality. Then they signed the informed consent and the interview began. The location and time of the interview were determined by the participants. Interviews were recorded with the tape recorder. Participants could stop the interview at any time, or delete part of their statements, and/or cancel the interview. This study was also approved by the Research Council of Tehran University of Medical Sciences and ethical approval was obtained under IR.TUMS.MEDICINE.REC.1398.214.

## Results

In this study, 21 people were interviewed who were 25-85 years old (49 years on average). Most (80%) of them were females and their education varied from below diploma to Ph.D., with different occupations. Except for one widow and one divorced, the rest were married and mostly in urban (66%) (table 1). 

**Table 1:** Interviewees’ demographic information

**No.**	**Gender**	**Age**	**Education**	**Occupation**	**marital status**	**Residential area**
1	Female	50	Associate degree	Teacher	Married	rural
2	Female	45	Diploma	Housewife	Married	Urban
3	Female	64	Diploma	Housewife	divorced	Urban
4	Female	58	Diploma	Tailor	Married	Urban
5	Female	29	B.C	Housewife	Married	Urban
6	Female	46	Diploma	Driver	Married	rural
7	Female	57	Diploma	Teacher	Married	Urban
8	Male	85	Under diploma	Retired	widow	rural
9	Female	25	B.C	Housewife	Married	Urban
10	Female	50	Diploma	Tailor	Married	Urban
11	Male	34	B.C	Employee	Married	Urban
12	Female	28	B.C	Housewife	Married	Urban
13	Male	39	M.D.	Employee	Married	rural
14	Female	31	B.C	Housewife	Married	rural
15	Female	64	Diploma	Housewife	Married	Urban
16	Male	73	Ph.D.	Pharmacist	Married	Urban
17	Female	63	B.C	Housewife	Married	Urban
18	Female	36	Diploma	Housewife (formerly employee)	Married	Urban
19	Female	45	Under diploma	Worker	Married	Urban
20	Female	47	Under diploma	Worker	Married	rural
21	Female	64	Illiterate	worker	Married	rural

The main concepts were extracted in the form of 71 initial codes. These codes then formed 21 subcategories based on similarities and differences and after removal or merging. Finally, seven categories of " responsibility", "patient's privacy", "informed consent", "respect and dignity of patient", "effective physician-patient communication", "trust in physician" and "conflict of interests" were identified, each had several subcategories (table 2).

**Table 2:** Initial codes, subcategories and categories of ethical issues in family medicine from the perspective of service receivers

**Category**	**Subcategory**	**Initial codes (example)**
Responsibility	Sufficient time for visiting the patient	Not allocating enough time/ enough and real time for patient/ optimal visit length based on referral type/ optimal visit length based on questioning/ fast visiting/ impatience
	Accurate diagnosis and prognosis	Accurate examining/ asking a lot of questions/ accurate diagnosis
	Consult with colleagues	Not-consulting with colleagues/ not-consulting with specialists
	Monitoring treatment process	Monitoring referrals/ not-monitoring/ possibility for calling after office hours/ non possibility for calling after office hours
	Responsibility about medical errors and/or mistakes	No mistaking/ not informing about the mistake/ mistake in diagnosis and treatment/ not responsibility about diagnosis and treatment
Patient's privacy	Providing safety for patient	Closing the door when visiting/ separation of doctor's room/ non separation of doctor's room/ accessibility to a private space/ separate examination
	Confidentiality and secrecy	Recording information by someone else/ opening the room's door
Informed consent	Emphasizing patient's requests	No asking for patient's need and problem/ not paying attention to patient's opinion
	Consult with patient about treatment process	Consultation of drug prescription
Respect and dignity of patient	Indiscrimination	Be fair with all patients/ patients with child playfulness/ patients with adults/ special attention to relatives
	Respect to patient	Be friendly/ standing or half raising to respect patient/ dignity in talking and behavior/ insulting/ crossness or aggression/ providing a calm space
Effective physician-patient communication	Comfort, intimacy, and communion	Comfort with a doctor of same gender/ comfort and intimacy/ relationship of father-child kind/ non comfort/ comfort with familiar doctor/ non comfort with specialist/ specialist' pride and prejudice/ economic perspective of healthcare operations/ no respecting to patient by specialist/ non possibility to talk with specialist/ stand silence and no explaining when no asking question by patient
	More accessibility to doctor	Possibility for calling after office hours/ non possibility for calling after office hours
	Paying attention to patient's confabulate and non-medical problems	Paying attention to patient's confabulate and non-medical problems/ non awareness of paying attention to non-medical problems when no discussing
	Mutual understanding	Tendency toward friendly and family relationship/ non tendency toward friendly and family relationship/ only by calling for questioning and guidance/ better understanding to improve trust and confidence
	Cultural matching	Better performance of religious doctors/ cultural, belief and age congruity
Trust in physician	Trust in doctor's academic knowledge	More trust to specialist/ busy clinic leads to trusts or distrust to family doctor (general doctor)
	Trust in doctor's behavior	Non moral trust/ trust to patient's behavior
Conflict of interests	Self-referral or referring to friends	Advice to refer an certain pharmacy/ advice to refer an certain laboratory/ advice to refer an certain specialist
	Bribery	Receiving bribe/ non receiving bribe
	Offering a gift	Non tendency to offer a gift/ tendency to offer a gift for appreciate/ tendency to offer a gift when important situation, illness or for discount

1) Responsibility

Responsibility is an important ethical issue that consists of five subcategories. 

A) Sufficient time for visiting the patients is important for them, and attention to it is one of the hallmarks of doctor's responsibility, according to interviewees' statements. Patients want more attention and a more accurate diagnosis from the doctor.

"My doctor says that the patient is always valuable to me and I need to examine her/his accurately. I clearly understand that he/she respects patients."- says a 50-year-old woman.

B) The patient's exact examination and diagnosis also indicate the doctor's responsibility, according to the interviewees.

"I feel my doctor isn't eager with my examination, especially that of last year; he/she was spending more time before that. When I speak to him/her after medical prescription, he/she works with his/her computer." – says a 29-year-old woman.

C) Almost all interviewees believe that their physician does not consult with colleagues or specialists. Patients often want their doctor to coordinate first before referring them to a specialist.

"Our doctor does not consult specialists. He/she only fills the referral form and insists on returning it back." - says a 34-year-old man.

D) Different opinions about monitoring of clinical care were raised. Some considered it acceptable, while some did not.

"Our family doctor is very good. My husband is afflicted with hypertension. Our doctor helps him in this case and always monitors his condition. In cases of delayed referral, the doctor wants his secretary to call my husband to refer to the office for checking hypertension, and he is always worried about him. Once my husband was suffering foot inflammation, and the doctor referred him to a few renal specialists, and then he/she asked us to submit the results for further investigation."- says a 58-year-old woman.

E) There was a different attitude about monitoring of medical errors and responsibility to them. Some believed that their physician did not make mistakes. Some were not aware of their doctors' mistakes. Some recalled their doctor's mistake. Some of the participants did not inform the doctor of his/her mistake, but the person who informed the doctor of his/her mistake was surprisingly confronted with resistance in accepting reality.

"Not about me. He/she has not done anything wrong. Every month, I go to the office one or twice to check the blood pressure. The doctor will record the results in the file, and he will inform us."- says an 85-years-old man.

"Our doctor has made a mistake twice, once about myself and once about my son. But we did not say anything to him/her. Indeed, I do not trust him/her anymore." says a 29-year-old woman.

2) Patient's privacy

The patient's privacy was important to the interviewees, observing it would increase the patient's satisfaction, comfort and security as well as trust in the doctor. It consists of two subcategories.

A) Providing a safe environment for the patient is a prerequisite for establishing appropriate doctor-patient communication as well as creating a quiet and secure environment for interviewing. Such an environment, however, was provided in most interview sessions. But in some cases, the failure to create it leads to not establishing the doctor-patient relationship and therefore a distrust.

"Recently, visits have been performed individually and in the closed room, while previously was in the open room, and people outside of the room were listening to us. The office space, of course, is a room, where the lower part is partitioned into two parts with a common roof with two other doctors talking together. Doctors hear each other, and I'm not satisfied with this space. I always try to talk quietly because the doctor nearby is a man."- says a 29-years-old woman.

(B) Confidentiality is also a prerequisite for the establishment of an appropriate doctor-patient relationship. In some cases, however, this is violated due to behavioral negligence and environmental defects. The presence of irresponsible and untrained people in the interview environment or the doctor-patient relationship, for instance, would lead to a violation of this case.

"During the examination, all information is recorded by a woman on the computer, and all of it is available in the next visit." - A 58-year-old woman says.

3) Informed consent

Informed consent - one of the most important indicators of respect to the patient's autonomy - is composed of two subcategories.

(A) Some patients emphasized the need for attention to their request by the physician and consultation on the treatment process is considered as their right.

"The doctor does not consult with me about the treatment process and thinks everything he prescribes is correct. My opinion is not important to him/her at all." - says a 45-year-old woman.

B) In Iran's ancient medicine, patients, especially the elderlies, did not care about their informed consent during the visit, so that some of them considered the doctor-patient consultation as illiteracy.

"I think these current doctors are illiterate; it seems they have not been trained because they ask me for medicine, and what should be performed." - says a 64-year-old woman.

4) Respect and dignity of patient

Patient dignity is another ethical issue extracted from interviews and has two subcategories.

A) Most patients were satisfied with the doctor's respect to the patient, and most of the dissatisfaction was about specialists. There were also some private cases of disrespect that could weaken or break the doctor-patient relationship.

"He/she is very polite and says hello to everybody and always has a smile on his/her lips. He/she always patiently listens to us." - A 34-year-old man says.

"We referred once at 7 pm. The door was closed but we could hear a sound from the room. The doorbell rang, and immediately he/she appeared shouted at us and said how much do you ring? And what happened? We said nothing, we just thought you were inside because we heard your voice. Since then I did not go to his/her office at all. I asked my husband to do my work." - says a 57-year-old woman.

B) Almost all patients did not share any experience of indiscrimination by the physician, and this was considered as the strength of the physician.

"My doctor's attitude is the same with all patients, and he/she respects everyone, whether an elderly woman who is walking hard or a playful child. He/she is very patient." - says a 50 year old woman.

5) Effective physician-patient communication

Establishing proper doctor-patient communication is a basis for building mutual trust. This category is divided into five subcategories.

A) Comfort, intimacy, and communion; most interviewees felt comfortable with their physician. Some of them chose the doctor of the same gender, and some of the relatives; in contrast, they could not establish a comfortable and intimate relationship with specialists for various reasons.

"I aimed at the family physician for the sake of comfort and intimacy, so a female doctor is my priority. I'm less comfortable with specialists because they are mostly males and I refer to them very little." - says a 29-year-old woman.

B) According to the Family Physician Program Executive Code, all patients should choose physicians who are active at a radius of 1.5 kilometers in order to increase accessibility. Some interviewees, however, have less accessibility due to reasons such as activity day's limits, daily hours of work, and the limitation of in-person contact, all of which were personal matters, not a systemic.

"There is no great distance between me and the doctor's office, and I can go there quickly. I'm suffering from hypertension, so the doctor gave me his phone number for an emergency." - says a 50-year-old woman.

C) Paying attention to patient's confabulate and non-medical problems will strengthen and deepen the physician-patient relationship and trust in the physician. Most of the interviewees protested to inattention to non-medical problems, and some of them considered the situation as contrary to the medical custom.

"No, I don’t have such an experience. Maybe this is the norm, that is, medical prescriptions and then a simple goodbye." - A 34-year-old man says.

D) Mutual understanding is another category that was extracted. The interviewees tended to know their physician well and establish a close relationship seeking for greater comfort. Well, this was varying; some were just looking for family and friendship, while some had to solve their medical problems, which required a close relationship with the ability to call for advice and guidance.

"Not so much that I want to contact with him/her outdoors, but I just want to establish a call, sometimes outside of regular working hours and some nights for emergency guidance. More than this isn’t acceptable. I expect him/her to answer my call outside the office hours at least until 11 pm." - says a 29-year-old woman.

E) Cultural matching can be a strong stimulus to establish a proper doctor-patient relationship, according to the interviewees, due to cultural, religious and age differences and different social classes.

"If we had more cultural and religious matching, or family or age relationship, just like a normal person, then our relationship would be stronger." - A 34-year-old man says.

6) Trust in physician

The patient's trust in the physician is the most important basis for the establishment and continuation of the doctor-patient relationship and even facilitates the process of disease improvement. Most interviewees had little confidence in their physician for a variety of reasons from the scientific level to ethical aspects. This category is divided into two subcategories.

A) Trust in doctor's academic knowledge; some patients believed that the busy office represents the doctor's high scientific level, but others considered this as a personal issue and believed that the doctor's level of education should be proven to them.

"I only refer to family physicians for simple illnesses like cold. For more severe ones, I will refer to a specialist because the general practitioner can't provide any special treatment plan. I suffer from kidney pain, for example; and in severe pain situations I refer to my own doctor." - says a 73-year-old man.

B) Trust in the doctor's behavior; most interviewees were satisfied with the physician's level of purity and chastity.

7) Conflict of interest

The interviews did not directly point the conflict of interest, but the interviewees - in explaining the doctor-patient relationship - pointed to cases that could clarify the existence of a conflict of interest or at least its position. This category has three subcategories.

A) The interviewees did not have full knowledge of self-referrals and did not consider it to be totally immoral. But the abundance of this issue was high and the interviewees have suspected the possibility of a secret relationship in this area (the probability of a conflict of interest) only when they had little confidence in their physician.

"Most of them are the same. They do not force the patient but tell him/her to go to a particular pharmacy. Some doctors are intelligent about this, but others are not. I think they have a contract with a particular pharmacy. 

When I asked my doctor, he/she says you can hardly find medicines elsewhere, but if you find you can take it." - says a 45-year-old woman.

B) No interviewees had ever bribed when they went to a family doctor, but some experienced it when they went to a specialist and/or had heard from their friends and relatives.

"Not about myself, but I heard something about others." - says a 25-year-old woman.

C) Some patients were faced offering a gift during their lifetime, but none of them considered it as an immoral act and only did it for appreciation sake.

"Not for me, but I love giving gifts, to encourage them, for example, buying flowers." - A 58-year-old woman says.

## Discussion

Content analysis of participants' interviews introduced "responsibility", "patient's privacy", "informed consent", "respect and dignity of patient", "effective physician-patient communication", "trust in physician" and "conflict of interests”. (table 2). 

When comparing the literature on family ethical issues in medicine with the results of this study, we reached to somewhat similar results, and in some different cases. (12-15, 19-30). Differences are many in foreign literature (12-14, 22-25, 27-29). They may be due to the socio-cultural differences of the communities surveyed, or less history of implementing the plan in Iran. Different moral issues and considerations - which participants did not mention - include resource allocation and health protection (12-14,19-22, 27-30), the beginning and the end of life (12-14, 19, 20, 22, 25, 30), social justice (19), ethics in research (12-14, 19, 22, 25), communications range and allowable expectations (12, 20), reproductive ethics and family planning (12-14, 20, 22), ethical issues of pregnant mothers, adolescents, the elderly and intellectually disabled people (12, 20, 22), legal aspects of family medicine (14), unpleasant and/or horrible news (14, 26), waiting lists (25), alternative decisions (12, 25]), the doctor's immoral behaviors include sexual contact, substance abuse, and more (12, 13), incompatible patients (27), referrals (27) and advanced directive (13). Referring to the fact that people usually point to issues that are involved, it is not uncommon to ignore the participants in this research with the above ethical considerations. Perhaps, if the type of study was different and the topics of ethics were presented as headlines, we would have come up with different answers. The following points and explanations are about the issues that the service recipients raised as family medical ethics issues. 

Matters related to responsibility, according to the description of their subcategories, in various texts entitled mistakes and medical abuses (12, 13, 19, 25), professional attitude and behavior (14), of physicians and students’ duties (22, 29, 30) and professional ethics (13). Therefore, responsibility with regard to its sub-categories is the doctor's responsibility to the patient's problems and issues. Unfortunately, the reversal of values has occurred, due to lack of proper notification and inappropriate supervision by the health system and lack of encouragement of responsible individuals and punishment of irresponsible doctors, so some patients interpret the complete duty performance by physicians, as limiting autonomy and neglecting the choice of patients. This problem should be corrected by the comprehensive and long-term training of doctors and patients as well as accurate monitoring and evaluation.

Consideration of patient privacy and confidentiality in most texts were considered as ethical considerations of family medicine (12-14, 19-20, 22, 26-27, 29-30). Nevertheless, it was very important to observe cultural issues in this regard. Open or closed door when examining the patient by the doctor of the opposite gender, for example, is interpreted differently at various cultural and religious levels. It is recommended to pay attention to the patient's presence or doctor's associate - under patient's consent - or the presence of the doctor of the same gender in order to observe the cultural and religious issues, besides considering opening or closing the room for patient's privacy. Or, on the other hand, recording patient information by someone else - which does not have a place in the patient-doctor relationship - is interpreted as facilitating and expediting the problems addressing from the perspective of patients, while it is, in fact, a kind of privacy violation.

Informed consent is also referred to in most texts of family medicine ethics (12-14, 19-20, 22, 25-27, 29-30). But the interviewees had a different, wide-ranging understanding of the concept of informed consent, from the patient that considered the physician-patient counsel, as a weakness, to the other one expected, the physician carries out all his desires and wishes, each one attributed the doctor who did not read their thinking to an immoral reputation. These are all due to the lack of training or inadequate teaching of ethical concepts, including informed consent to health services providers and receivers, as well as inadequate media advertising on corresponding service providing plans.

Considering the patient dignity and its subcategories have been referred in various texts under different titles, including the values of patients (14, 22, 29) and justification with patients (13, 19, 20, 28). However, this is often a background for any kind of human behavior and any medical communication, not just in the field of family medical ethics. In most of the texts studied, effective doctor-patient communication has been mentioned (12-14,19-27, 29-30). But the important issue was the levels of communication, because the interviewees were at a different age, cultural, social, and religious levels. This demonstrates the sensitivity and difficulty for physicians to establish a proper relationship with patients and to satisfy them. Extensive efforts and practical training, therefore, should be undertaken in this regard.

Addressing some issues, however, needs to modify systemic processes. In Iran's cultural and social space, for example, both male and female doctors must be present at the clinic, with the aim of increasing patients' comfort when expressing their own problems and the religious issues regarding contact with a doctor of the opposite gender. This increases the level of comfort and mutual trust and hence improves the patients' health. On the other hand, public medicine courses emphasize limiting communication that leads the level of patient-doctor relationship to decrease dramatically. Or people who are willing to have a friendly and family relationship out-of-work environment, are often looking for medical support and guideline at out-of-office hours, not a friend or partner. This indicates that the level of communication required or requested by patients should go beyond the current level and this need should be meted to increase patient satisfaction.

In different texts, patient's trust in doctor has been referred to a lesser extent (19, 20), while honesty - as a prerequisite for trust - has gained a double emphasis (29, 30), because trust is usually the result of a long relationship along with different ethical issues. But due to limitations in choosing a doctor in a family medicine plan, most choices are not based on full knowledge, which results in a low level of trust resulting from a short-term relationship. Also, due to the culture of professionalism in society - with decades of history - family physicians are often regarded as illiterate by the community, which leads to a reduction in mutual trust level.

Conflict of interest has also been studied in most texts (12-14, 19-27, 29-30). However, various conflicts of interest and their aspects have been presented due to the cultural and social differences between societies. For example, communication with pharmaceutical industries is important in western societies (13, 19), while interviewees considered self-referral to be one of the examples of conflict of interest that could be due to low information on other conflicts of interest and/or regarding other things immoral. And, undoubtedly, this requires more extensive research.

In general, the ethical considerations of family medicine program can be categorized into two areas of program specifications and how it is implemented. The findings of this study and the topics discussed may include one or both areas because people could not differentiate between the program and its implementation. Therefore, to plan and correct problems, an issue and its scope should be considered. Thus in some cases, modifications should only be made in the program or in processes. But while the problem is in the implementation of the program in a particular region, in addition to paying attention to the executor performance, the conditions of the environment must be examined, so that in cases where the cultural and social problems hindered the implementation of the program, comprehensive and long-term training programs can fix the bugs. These training programs for executives include educational and ethical guidelines and curricula that should be prepared and reviewed according to the culture of the country and the region of implementation.

Due to the low resources and literature on the family medicine ethical considerations, it is one of the strengths of this study to carry out studies of this kind which, on the one hand, are in accordance with Iranian and Islamic culture and, on the other hand, address the needs and desires of the recipients of the service. The results of such a study can identify shortcomings in the design and implementation of this program and suggest ways to improve it in the future, and, as the people's ideas are used in designing these strategies, it would also satisfy the popular satisfaction and acceptance.

This study had some limitations. One is the generalization of qualitative study results. Regardless of geographic constraints - which may reduce cultural and social diversity as well as opinion limitations - we have tried to study participants of the different ages, educational levels, occupations, and social classes. Another is that majority of participants were females that is consistent with the statistical population of the referrals to the family medicine. Most family physicians are active at a time when men are working. And the majority of referrals are children. So, more women referring to the clinic and hence participating in the study than men are expectable. Another limitation was the cultural problems of the Iranian health system. Consequently,people were not familiar with the characteristics of a good physician. Undoubtedly, we are facing lack of awareness and the need for people to be educated by members of the health system and the media about this and other regards.

In conclusion based on the findings of this research, there are differences in ethical issues between patient opinions and the designers of family medical ethics curriculum. However, patient's satisfaction and enhancement of mutual trust are essential. Patient comments should be considered when providing ethical guidelines. Particularly when the family medical plan is to be created in a cultural context that is fundamentally different from the culture of the leading countries in this regard.

Based on the results of this study, we can achieve a better understanding of patient perceptions about family medicine ethics, and this enables us to better recognize the weaknesses of family physicians and the health system to strengthen mutual trust and doctor-patient relationship. We can also design new ethical guidelines in family medicine and to provide them in educational levels.
